# Are Pandemics Gender Neutral? Women's Health and COVID-19

**DOI:** 10.3389/fgwh.2020.570666

**Published:** 2020-10-19

**Authors:** Hannah Simba, Silindile Ngcobo

**Affiliations:** ^1^Department of Global Health, Faculty of Medicine and Health Sciences, Stellenbosch University, Cape Town, South Africa; ^2^Independent Researcher, Cape Town, South Africa

**Keywords:** COVID-19, pandemic, women's health, outbreaks, gender inequality

## Abstract

In December 2019, coronavirus disease 2019 (COVID-19) emerged as a health crisis in Wuhan, China, and was later declared by the World Health Organization (WHO) as a Public Health Emergency of International Concern. As it spread and its death toll increased, on the 11th of March 2020 it was declared a pandemic at 4,369 deaths worldwide, and cases and deaths have since surged. With gender disparities already known to leave women and their health at the margins of society during outbreaks, it is important to understand how COVID-19 affects women's health. In this article, we discuss how the COVID-19 pandemic can create vulnerabilities for women and their health and further exacerbate long-existing inequalities and social disparities. These include gender-based roles, economic and food security, violence, work pressure, and access to health and healthcare facilities. These issues have significant repercussions on the physical and mental health of women. To focus our lenses on these issues, we draw lessons from three specific examples of past outbreaks: 1918 Flu pandemic, Zika virus disease, and Ebola virus disease. We conclude by stating how public health responses and strategies for COVID-19 can be inclusive to women's health.

## Introduction

At present, the world is in the middle of a coronavirus disease 2019 (COVID-19) outbreak, declared a pandemic on the 11th of March 2020 by the World Health Organization (WHO). The first outbreak was confirmed in Wuhan, China, on the 31st of December 2019, and to date, cases have been reported in at least 188 countries (1). Infected individuals may be asymptomatic or have pre-symptomatic infection, while symptomatic presentation ranges from mild to severe respiratory distress (1, 2). With no existing vaccine therapy, treatment options are limited to broad-spectrum antivirals and management of symptoms. Clinical outcomes are dependent on the patient's immune system, chronic comorbidities, and age, with the elderly holding the highest risk (1). In several countries, measures to control transmission have been implemented at an unprecedented scale. These measures include self-isolation for the infected, quarantine for the exposed, wearing of masks in public places, local and international travel restrictions, and closure of schools and businesses (3). Currently, sex disintegrated data, although incomplete, shows higher numbers of COVID-19 cases in women compared to men, with higher mortality rates in men (4). Understanding the gendered impact of COVID-19 and exploring how it affects women will allow for effective and equitable pandemic responses.

## Gender Disparities and COVID-19

The differences in how women fare during a pandemic compared to men are largely due to long-existing inequalities and social disparities, which are exacerbated by the pandemic, rather than biology (5). Inequalities created and compounded by outbreaks leave women in a more vulnerable position (6). To put it into perspective, globally, women form 70% of the healthcare and social services workforce (7). This automatically puts them at the frontline during a pandemic response, and thus, they face a high risk of infection. Data compiled by the WHO from over 104 countries (2000–2018) showed that women constituted as the majority of the nursing personnel in the African region (65%), region of the Americas (86%), Eastern Mediterranean region (70%), European region (84%), South-East Asia region, (79%), and Western Pacific region (81%) (7). The female physician population varied in these regions ranging from 28% (Africa Region) to 53% (European region). In the Hubei province of China, more than 90% of the health workforce was reported to be women (8). In a study investigating mental health outcomes of frontline healthcare workers in China, women and nurses were at a higher risk of developing unfavorable mental health outcomes including depression, anxiety, insomnia, and distress (8). Furthermore, personal protective equipment (PPE) shortage for healthcare workers, together with the gendered nature of the healthcare care workforce, puts the women at even higher risk of infection (9). PPE shortage has been reported in several countries since the COVID-19 outbreak. It is important to emphasize that PPE shortages endanger the health of all healthcare workers.

Surviving a pandemic for women means more than just surviving the disease, as there are threats beyond the risk of infection. It is true that during crises, epidemics and pandemics, women tend to take up more caregiver responsibilities than usual, often at the expense of their health (10). Particularly in the COVID-19 crisis, wherein some instances families have to stay at home while self-isolating or during movement restrictions, women can be overworked and overstretched as they take on more domestic care. This increasing burden of care can also take time away from paid work (6). It is therefore clear that to recognize the different patterns of exposure between men and women, understanding societal norms is imperative. The responsibility of taking care of the sick also often falls more on the women at home (11). In some cases, the women at the frontline of pandemic responses have a double-barrel role of being the caregivers both at work and at home, putting women at a higher risk of infection. A gender analysis of reporting media from four countries (Sri Lanka, Malaysia, Vietnam, and Australia) during the COVID-19 pandemic reported intersections between Covid-19 and gendered burdens, particularly in frontline work, unpaid care work, and community activities (visiting the sick, cooking, and cleaning) (12).

During pandemics, women are at a greater risk of more violence and abuse (13). The lockdown and isolation policies implemented in many countries put women at a higher risk of domestic and sexual abuse as they are likely to spend more time with their abusers (11, 13). The need for protection of women against abuse is therefore heightened during the COVID-19 pandemic. Studies looking into the surveillance and evaluation of effective interventions for those at risk of domestic violence during the pandemic are still lacking in literature (13). An increase in teenage pregnancies may also be experienced, due to several factors including sexual violence and negative coping strategies. The need for financial support can also increase exploitative relationships resulting in more teenage pregnancies (11).

Health seeking behavior and access to health care may also affect access to treatment. While in most high-income countries women are more likely to utilize healthcare services than men (11), in some societies women are less likely to seek healthcare services on their own due to social norms or if the healthcare provider is male (5). It has been shown that poor women are less likely to seek healthcare services (5). Furthermore, research on whether women face specific constraints to access healthcare services including the level and type of care during the COVID-19 pandemic needs to be investigated.

The COVID-19 crisis poses a threat to several aspects of women's rights, including reproductive rights, economic rights, and other freedoms. Sexual and reproductive health services remain important even during pandemics. In some countries, however, these become overlooked as funding becomes diverted to pandemic responses. This has dire health (including mental health) consequences for women needing these services. It is projected that due to COVID-19, millions of women and girls may be deprived of family planning services (11). Women's rights and economic gains have been affected by COVID-19. The changes in power relations between men and women during a crisis expose women's vulnerabilities and increases burdens. Generally, during a crisis, women's decision-making power in the home often regresses, as reported in studies done in Zimbabwean and Ethiopia (14). Additionally, in Mali and Niger, women are the first to lose land and income during a crisis (14). This pattern will likely be repeated during the COVID-19 pandemic, leaving a lot of women disenfranchised and rolling back women's rights.

The world food program reported that the number of people who will face a food crisis will likely double because of COVID-19 and warned of a hunger pandemic (15). For women and girls, this could have even worse implications as they already constitute 60% of those facing a food crisis and 76% of the displaced population worldwide (14). Food security for women is therefore at great risk, with more women likely to face a food crisis due to COVID-19. Furthermore, women also face the brunt of food insecurity as in most households the responsibility of feeding the family falls on them (14). Shortages of food in the home means women will more likely sacrifice the food that is available for their children and families by eating less and eating last, resulting in malnutrition. This generally makes more women to be more susceptible to non-communicable diseases and other diseases.

Current data on maternal health has not shown maternal–fetal transmission of COVID-19 (16, 17). This is in contrast to the experience of two other known pathogenic coronaviruses, severe acute respiratory syndrome (SARS), and Middle East respiratory syndrome (MERS), which have been reported to increase maternal morbidity and mortality. Pregnant women have also been reported to not be at a greater risk for contracting COVID-19, compared to non-infected pregnant women (18). However, the immune system is known to experience suppression in normal pregnancy, resulting in increased susceptibility to infection; hence, pregnant women are still a vulnerable patient population (17). Guidelines on the management of pregnant women during the COVID-19 pandemic are continuously being updated. More follow-up and bigger studies on pregnant women and infants with COVID-19 are needed to evaluate their health and safety. Additionally, the inclusion of women in clinical trials for COVID-19 vaccines is imperative.

It is important to reiterate that women's issues stated here did not suddenly appear during this COVID-19 pandemic but have been or will be compounded by it. These issues have a direct and indirect influence on several aspects of women's health, including putting them at a greater risk of COVID-19 infection, worsening already existing diseases, and lastly making them more susceptible to new ailments of physical and mental health. Is COVID-19 gender neutral? No. The gendered burden of COVID-19 is clear and undeniable. Lessons from past outbreaks can shed light on how to better prepare for an inclusive COVID-19 response system.

## Examples From the Past

### Zika Virus Disease

The first human case of the Zika virus (ZKV) disease was reported in 1952 (19). In 2015, an outbreak began in Brazil and spread to parts of North and South America, Southeast Asia, and several Pacific Islands (20). The outbreak took a toll on pregnant women. As they delivered, a pattern of newborns presenting with congenital defects, collectively known as Congenital Zika Syndrome (CZS), such as microcephaly was observed (21). Some women experienced preterm births, stillborn births, and miscarriages (22–24). In Brazil, the epicenter, between 5 and 15% of newborns to infected mothers developed microcephaly and on the basis of the clusters known, microcephaly was declared as a Public Health Emergency of International Concern (25, 26). National governments further advised that women of reproductive age delay pregnancy and avoid unprotected sexual intercourse. Contraception was provided as an alternative despite inadequate health education on where and how the women could access family planning services (27, 28). Subsequently, the WHO's interim guide recommended abstinence and irrationally advised on guarding against mosquito bites as a prevention strategy as the women bear a large responsibility of conducting vector control activities (29). These recommendations infringed on their autonomy and SRH rights and further suggested that women bear the sole responsibility of managing their risk profiles during outbreaks, without supporting resources.

Power dynamics granted women with lesser power in decision-making (30, 31). Abortion is still not accessible in some countries as it is either criminalized or available in restricted circumstances. For example, in African countries such as Angola and Latin American El Salvador (one of the epicenters), abortion is criminalized, while in Brazil it is restricted to anencephaly. Resultantly, multiple El Salvadorean women were sentenced with abortion charges during this outbreak, regardless of whether it was unclear cases of miscarriages or induced abortions (26). Others had unsafe abortions while others faced unprepared for financial, physical, and psychological responsibilities of raising CZS children after pregnancy with limited support (26). These experiences have been implicated in placing women at a higher risk for mental illnesses such as anxiety and depression (32).

### Ebola Virus Disease

The 2014–2016 West Africa Ebola virus disease (EVD) outbreak was the most widespread since the virus's discovery in 1976 (33). It highlighted the consequences of neglecting gender-inclusive perspectives during a crisis. Gender is a determinant of health, and gender roles contribute substantially to transmission. They influence where women and men spend most of their time, what infectious agents they are exposed to, and duration and frequency of exposure (5). During this outbreak, risk of transmission was high among those caring for the sick at home (PPR 13.33) and conducting funeral activities (PRR^*^ 4.8) (34). These are two gender roles that sociocultural norms dictate for women In West Africa. No biological sex differences have been implicated to EVD infection vulnerability, while several sociocultural and healthcare factors have been reported to have increased the risk of infection (33).

In low-and-middle-income-countries (LMICs), as in West Africa, health systems are overburdened and resilience against outbreaks is low (35, 36). This is characterized by inaccessible healthcare service, lack of support for a diverse population, and challenges with identifying and isolating health threats while maintaining its core functions. Limited resources are also diverted toward emergency responses (37, 38). In Sierra Leone, preexisting lack of resilience in the health system has been reported to have contributed to reduced utilization of healthcare services, including maternal and newborn health (MNH) services. Pregnant women lacked trust in the low-resilient health system and were resultantly reluctant to access routine healthcare services, concerned about contracting the infection. Structural barriers (e.g., public transport utilization also influenced access to healthcare). Subsequently, this delayed maternal and neonatal health care, indirectly affecting maternal, stillbirth, and neonatal mortalities (39–41). The United Nations Fund for Population Activity (UNFPA) reported that pregnant women in labor were concerned about the competency of their healthcare providers and lack of protection in preventing infection (42). It is since been predicted, through mathematical models, that a 50% reduction in accessing healthcare services potentially exacerbated mortality rates for HIV/AIDS, tuberculosis, and malaria with 2,819 excess deaths in Sierra Leone, 6,269 in Guinea, and 1,535 in Liberia (43). These are infectious diseases that also affect women.

During this outbreak, delayed healthcare was also experienced as a consequence of a broad and vague EVD case definition. There was therefore confusion around its application. In this, unexplained bleeding and spontaneous abortion were used as markers for isolation to Ebola Treatment Centers (ETCs). These markers could not be differentiated from miscarriages. Furthermore, unexplained bleeding is a sign of several obstetric complications. As a result, this was a contributing factor to pregnant women's reluctance in seeking healthcare. They also feared being wrongly isolated to ETCs (39, 41, 44). The overall reluctance in healthcare-seeking behavior among women also meant that sexual assault victims were also compromised with post-rape care.

It is worth noting that women play large roles in agriculture and are affected during restricted trade. Herman reported in 2015 that Sierra Leone's gross domestic product (largely supported by agriculture) dropped from 8.9 to 2.0% due to restricted trade during the EVD outbreak (45). Such repercussions affect women's jobs and limit women's participation in the economy.

### 1918–1919 Influenza Pandemic

It is just over 100 years since the world's deadliest pandemic, 1918–1919 influenza (flu) with a 50 million estimated death toll (46). In South Africa, about 5% of the total population perished, and right across Africa food security and transport were disrupted (46, 47). The pandemic emerged at a time of underdeveloped medical care globally; hence, incomplete epidemiologic data to date and various challenges were encountered. In America, for instance, the pandemic emerged at a time of war distress, 4 years into World War I (WWI). Public health officials implemented response strategies i.e., isolation and quarantine, to curb transmission (48). This meant more responsibility for women with caregiving roles.

In America, the pandemic distress contributed to a labor shortage (48). The shortage prompted socioeconomic transformation and more women entered the workforce to fill labor gaps. They took up work in the frontlines, while they still had caregiving and childbearing responsibilities at home. This also happened while the women were in movements advocating for their right to vote (46, 49, 50). Their responsibilities and roles heightened, while the risk of infection threatened their health.

## What Do We Learn From the Past and What Does this Mean for COVID-19?

Pandemics exacerbate existing gender inequalities. As seen in the three examples, gender norms, unprepared health systems, inaccessible healthcare services, and power dynamics increase women's vulnerabilities during a crisis. Therefore, pandemics are not gender neutral. In the EVD outbreak, gender roles exposed women to a high risk of infection through caregiving and burial activities. The low-resilient health systems led to women not being able to access healthcare services timely. Unprepared health systems resulted in the neglect of women's SHR services while funds were being diverted toward emergency responses. Subsequently, lack of clear, accurate, and effective communication in responses further compounded these challenges (34, 38, 41). In the ZKV outbreak, power dynamics favored women's exclusion in decision-making, resulting in their autonomy being infringed and SHR rights undermined. Lack of various forms of support for mothers post-pregnancy also became a challenge (26, 28, 30). The frontline healthcare brigade is largely made of women who risk their lives. This was also seen during the 1918–2019 flu outbreak with American women filling labor gaps to curb the pandemic (46, 48). These had additional caregiving and childbearing responsibilities, hence a heightened workload.

Goal 5 of the 2030 Agenda for Sustainable Development Goals (51) aims to achieve gender equality and empowerment women by 2030. COVID-19 public health response strategies should, therefore (52):

- Address gender norms and the need for shared responsibilities at home and in the workplace.
- Prioritize frontline workers' health, including mental health for all women.
- Integrate SRH rights for all women and put in place monitoring strategies.
- Provide accurate and accessible family planning education and all healthcare services.
- Incorporate and keep surveillance and protection systems for gender-based violence victims.
- Be sensitive to the women who are in informal labor because, in LMICs, women also dominate this sector (as mentioned for Sierra Leone during EVD outbreak). There must be clear plans of action to assist these women when there are movement restrictions and there are economic repercussions.
- Appoint women in leadership and management positions for national task teams and global organizations.
- Prioritize and support ongoing scientific research, collaboration, and provide funding for it.

## Conclusion

In conclusion, outbreaks exacerbate already existing gender inequalities. In the COVID-19 pandemic; women's health needs to be prioritized as women are more vulnerable during this time—as frontline healthcare workers, as primary caregivers at home, as informal sector laborers, and as citizens needing access to healthcare facilities. Sexual and reproductive health rights and access to healthcare should not be neglected during this time. Women and women's perspectives are needed when making decisions for pandemic planning and strategies. Gender informed responses and strategies addressing the gender inequalities that persist during outbreaks must be the norm.

## Data Availability Statement

The original contributions presented in the study are included in the article/supplementary material, further inquiries can be directed to the corresponding author/s.

## Author Contributions

SN and HS conceptualized the idea for the research, performed literature searches, drafted the manuscript, contributed to the critical revision, and intellectual input of the manuscript. All authors contributed to the article and approved the submitted version.

## Conflict of Interest

The authors declare that the research was conducted in the absence of any commercial or financial relationships that could be construed as a potential conflict of interest.
